# Factors Associated With Serological Response to SARS-CoV-2 Vaccination in Patients With Multiple Sclerosis Treated With Rituximab

**DOI:** 10.1001/jamanetworkopen.2022.11497

**Published:** 2022-05-11

**Authors:** Andreas Tolf, Anna Wiberg, Malin Müller, Faisal Hayat Nazir, Ivan Pavlovic, Ida Laurén, Sara Mangsbo, Joachim Burman

**Affiliations:** 1 Department of Medical Sciences, Uppsala University, Uppsala, Sweden; 2 Department of Immunology, Genetics and Pathology, Uppsala University, Uppsala, Sweden; 3 Department of Pharmacy, Science for Life Laboratory, Uppsala University, Uppsala, Sweden

## Abstract

**Importance:**

B-cell–depleting monoclonal antibodies are widely used for treatment of multiple sclerosis but are associated with an impaired response to vaccines.

**Objective:**

To identify factors associated with a favorable vaccine response to tozinameran.

**Design, Setting, and Participants:**

This prospective cohort study was conducted in a specialized multiple sclerosis clinic at a university hospital from January 21 to December 1, 2021. Of 75 patients evaluated for participation who received a diagnosis of multiple sclerosis with planned or ongoing treatment with rituximab, 69 were included in the study, and data from 67 were analyzed.

**Exposures:**

Sex, age, number of previous rituximab infusions, accumulated dose of rituximab, previous COVID-19 infection, time since last rituximab treatment, CD19^+^ B-cell count before vaccination, CD4^+^ T-cell count, and CD8^+^ T-cell count were considered potential factors associated with the main outcome.

**Main Outcomes and Measures:**

Serological vaccine responses were measured by quantitation of anti-spike immunoglobulin G (IgG) antibodies, anti–receptor-binding domain (RBD) IgG antibodies, and their neutralizing capacities. Cellular responses to spike protein–derived SARS-CoV-2 peptide pools were assessed by counting interferon gamma spot-forming units in a FluoroSpot assay.

**Results:**

Among 60 patients with ongoing rituximab treatment (49 women [82%]; mean (SD) age, 43 [10] years), the median (range) disease duration was 9 (1-29) years, and the median (range) dose of rituximab was 2750 (500-10 000) mg during a median (range) time of 2.8 (0.5-8.3) years. The median (range) follow-up from the first vaccination dose was 7.3 (4.3-10.0) months. Vaccine responses were determined before vaccination with tozinameran and 6 weeks after vaccination. By using established cutoff values for anti-spike IgG (264 binding antibody units/mL) and anti-RBD IgG (506 binding antibody units/mL), the proportion of patients with a positive response increased with the number of B cells, which was the only factor associated with these outcomes. A cutoff for the B-cell count of at least 40/μL was associated with an optimal serological response. At this cutoff, 26 of 29 patients (90%) had positive test results for anti-spike IgG and 21 of 29 patients (72%) for anti-RBD IgG, and 27 of 29 patients (93%) developed antibodies with greater than 90% inhibition of angiotensin-converting enzyme 2. No factor associated with the cellular response was identified. Depending on the peptide pool, 21 of 25 patients (84%) to 22 of 25 patients (88%) developed a T-cell response with interferon gamma production at the B-cell count cutoff of at least 40/μL.

**Conclusions and Relevance:**

This cohort study found that for an optimal vaccine response from tozinameran, rituximab-treated patients with multiple sclerosis may be vaccinated as soon as possible, with rituximab treatment delayed until B-cell counts have reached at least 40/μL. An additional vaccination with tozinameran should be considered at that point.

## Key Points

**Question:**

Are certain demographic or clinical factors associated with a favorable vaccine response to tozinameran among rituximab-treated patients with multiple sclerosis?

**Findings:**

In this cohort study of 67 patients with multiple sclerosis, among several assessed disease- and treatment-associated factors, B-cell count was the only factor associated with the serological response to tozinameran, whereas the cellular response was not associated with any of the investigated factors. B-cell counts of at least 40/μL were identified as the optimal cutoff to achieve a serological response in most patients.

**Meaning:**

These results suggest that rituximab-treated patients with multiple sclerosis may be vaccinated with tozinameran as soon as possible, with rituximab treatment delayed until B-cell levels reach 40/μL, when an additional vaccine dose should be considered.

## Teaser

This cohort study conducted at a specialized multiple sclerosis (MS) clinic investigates factors associated with serological response to tozinameran in patients with MS receiving rituximab.

## Introduction

B-cell–depleting monoclonal antibodies are widely used for treatment of multiple sclerosis (MS) and for many other conditions. The treatment is commonly administered at outpatient clinics in intervals of 3 to 18 months, depending on the indication and duration of treatment, leading to a prolonged depletion of B cells. Although rarely performed in clinical practice, B-cell counts may be measured with flow cytometry, which can be used to monitor B-cell–depleting therapies over time.

An adverse effect of B-cell–depleting therapy is that the protective humoral immune response that normally occurs after infections and vaccinations is weakened or absent.^1,2,3,4,5,6^ For many patients with chronic diseases, such as MS, this poses a challenge for the treating physician when vaccinations need to be administered after the initiation of B-cell–depleting therapy.^7^ This problem was brought into focus in connection with the COVID-19 pandemic caused by SARS-CoV-2, when several studies showed a higher risk of severe COVID-19 among people with MS and concomitant anti-CD20 therapy.^8,9^ In addition, ongoing B-cell–depleting therapy has been associated with a decreased humoral immune response following vaccination against SARS-CoV-2.^10,11,12,13^

Some authors and guidelines recommend vaccinations being given at the earliest 3 months, but preferably 6 months, after the last administration of a B-cell–depleting agent and that at least 4 weeks pass from vaccination until the next administration.^10,14^ Others have suggested that vaccinations should be postponed until the CD19^+^ B-cell count has reached at least 20/μL,^15^ but robust data to support these recommendations are lacking. Although a body of literature on humoral and T-cell responses after vaccination against SARS-CoV-2 in patients with MS treated with B-cell–depleting agents emerged in the previous year,^11,12,13,16,17^ it is still unknown which factors are most important for an adequate vaccine response. The purpose of the present study was to investigate which factors were associated with a favorable vaccine response after vaccination with tozinameran for protection against COVID-19 disease.

## Methods

### Study Design and Setting

The study was performed as a single-center, prospective, observational cohort study at the outpatient clinic of the Department of Neurology at Uppsala University Hospital, Sweden, and followed the Strengthening the Reporting of Observational Studies in Epidemiology (STROBE) reporting guidelines.^18^ The study was approved by the Swedish Ethical Review Authority and was performed in concordance with the Declaration of Helsinki (1964).^19^ Oral and written consent were obtained from each participant. No one received compensation or was offered any incentive for participating in this study.

### Standard of Care

All individuals undergoing treatment with rituximab for MS were offered vaccination against SARS-CoV-2 with tozinameran. Patients were recommended for vaccination if at least 6 months had passed since their last infusion of rituximab and if their B-cell counts were at least 20/μL. If the B-cell count was lower than 20/μL, patients were offered to postpone their vaccination (and rituximab treatment) and a new flow cytometry was performed 2 months later. This was repeated up to 3 times, and then vaccination was recommended regardless of the B-cell count. All patients could proceed with vaccination at any time regardless of B-cell count, and some patients opted for vaccination right away. The Pfizer-BioNTech COVID-19 vaccine tozinameran was used, with 2 doses given at an interval of 3 weeks. Treatment with rituximab was administered 6 weeks after the second vaccination dose or later, depending on the prescribed infusion interval. Patients were encouraged to report any suspected adverse effects of the vaccination or symptoms suggestive of MS-associated disease relapse.

### Participants

Eligible patients (received a diagnosis of MS and had ongoing or planned rituximab treatment) were followed up at the Department of Neurology, Uppsala University Hospital, Sweden. Two doses of tozinameran were administered to all patients from January 21 through August 19, 2021. A blood sample was drawn prior to the first vaccination dose and another sample was drawn 6 weeks after the second vaccination dose. The last follow-up of patients was conducted on December 1, 2021; median (range) follow-up from the first vaccination dose was 7.3 (4.3-10.0) months.

### Factors Potentially Associated With the Vaccine Response

Data on sex, age at vaccination, number of previous infusions of rituximab, accumulated dose of rituximab, previous positive SARS-CoV-2 polymerase chain reaction test result, time since last treatment with rituximab, CD19^+^ B-cell count prior to vaccination, CD4^+^ T-cell count, and CD8^+^ T-cell count were collected from medical records at the time of the first vaccination dose. The COVID-19–associated factors were assessed again when the blood sample was drawn after vaccination.

### Outcomes

The primary outcomes were serological response and cell-mediated immune response after vaccination. Serological immune response was defined as 264 binding antibody units (BAU)/mL for anti-spike (anti-S) IgG and 506 BAU/mL for anti–receptor-binding domain (RBD) IgG (using the World Health Organization’s international standard, National Institute for Biological Standards and Control code 20/136), based on a demonstration of 80% vaccine effectiveness against primary symptomatic COVID-19 infection in a previous study.^20^ Cell-mediated immune response was defined as interferon gamma (IFN-γ) spot-forming units (SFUs) exceeding 4 when stimulated with an in-house peptide pool and exceeding 10 SFUs when stimulated with a commercial peptide pool. The cutoffs were based on receiver operating characteristic curves, where the highest sensitivity and specificity for the respective assay were chosen. If a patient sample exceeded the cutoff before vaccination, a 2-fold increase in SFUs was required after vaccination for the cell-mediated immune response to be considered positive.

### Evaluation of Humoral Immune Response After SARS-CoV-2 Vaccination

Antibodies against nucleocapsid, RBD, and spike were determined with a V-PLEX SARS-CoV-2 Panel 2 (IgG) kit (Meso Scale Diagnostics). A V-plex SARS-CoV-2 Panel 2 (ACE2) kit was used to determine the antigen neutralization capacity in an angiotensin-converting enzyme 2 (ACE-2) competitive assay.

### Evaluation of Cell-Mediated Response After SARS-CoV-2 Vaccination

Following stimulation with either a commercial peptide pool (Mabtech) containing 100 peptides derived from the human SARS-CoV-2 spike protein or an in-house–developed peptide pool of 8 peptides derived from the spike protein^21,22^ (eTable 1 and eTable 2 in the Supplement), the number of SFUs was determined with a Mabtech IRIS FluoroSpot reader, and spots were analyzed using Mabtech Apex software, version 1.1. A detailed description of this assay is available in the eMethods in the Supplement.

### Statistical Analysis

Graphs and statistical analyses were generated with Prism 9 (GraphPad). The Wilcoxon matched-pairs signed rank test was used for comparisons between paired samples, and the Mann-Whitney test was used for nonpaired comparisons. Correlations were described with the Spearman rank coefficient of correlation. Receiver operating characteristic curves were generated to assist in selecting the optimal cutoffs. A multiple logistic regression model was used to describe B-cell recovery. Multiple linear regression models were used to investigate which factors were associated with anti-S IgG levels and IFN-γ SFU levels. A 2-tailed *P* < .05 was considered statistically significant. For details see the eMethods in the Supplement.

## Results

### Participants

Of 75 individuals assessed for eligibility in the study, 69 were included, and data from 67 were analyzed (eFigure 1 in the Supplement). Of them, 60 had ongoing rituximab treatment and 7 were rituximab-naive. For 60 patients with ongoing rituximab treatment, the mean (SD) age was 43 (10) years, 49 were women (82%), 11 were men (18%), 49 (73%) had relapsing-remitting MS, and the median Expanded Disability Status Scale score was 2.0 (range, 0.0-8.0). The median (range) dose of rituximab was 2750 (500-10 000) mg during a median (range) time of 2.8 (0.5-8.3) years. Other descriptive data for the participants are presented in Table 1.

**Table 1.** Patient Demographic and Clinical Data at the Time of the First SARS-CoV-2 Vaccine Dose

Characteristic	No. (%)	
	Ongoing rituximab (n = 60)	Anti-CD20 naive (n = 7)
Age, mean (SD), y	43 (10)	36 (10)
Sex		
Female	49 (82)	5 (71)
Male	11 (18)	2 (29)
Time since MS diagnosis, median (range), y	9.4 (1.2-28.9)	0.2 (0.0-9.1)
MS phenotype		
PPMS	2 (3)	1 (14)
RRMS	44 (73)	5 (71)
SPMS	14 (23)	1 (14)
Time since SPMS diagnosis, median (range), y	6.3 (0.13-10.0)	NA
EDSS score, median (range)	2.0 (0.0-8.0)	2.0 (0.0-5.5)
Disease-modifying treatment prior to rituximab		
0	8 (13)	5 (71)
1	27 (44)	1 (14)
2	14 (23)	1 (14)
3	9 (15)	0 (0)
4	2 (3)	0 (0)
Interferon-β	40 (67)	1 (14)
Dimethyl fumarate	8 (13)	2 (29)
Fingolimod	2 (3)	0 (0)
Glatiramer acetate	16 (27)	0 (0)
HSCT	2 (3)	0 (0)
IVIG	10 (17)	0 (0)
Natalizumab	8 (13)	0 (0)
Ocrelizumab	1 (2)	0 (0)
Teriflunomide	2 (3)	0 (0)
Ongoing rituximab therapy		
Infusion interval of 6 mo	52 (87)	NA
Infusion interval of 12 mo	8 (13)	NA
Duration of treatment, median (range), y	2.8 (0.5-8.3)	NA
Accumulated dose, median (range), mg	2750 (500-10 000)	NA
CD19^+^ B-cell count before vaccination		
Median (range), /μL	38 (0-348)	NA
≥40	29 (48)	NA
30-39	8 (13)	NA
20-29	10 (17)	NA
10-19	8 (13)	NA
0-9	5 (8)	NA
Time from infusion to test before vaccination, median (range), wk	34 (25-79)	NA
Time from test before vaccination to test after vaccination, median (range), d	9 (0-258)	NA
Participants with ≥1 positive SARS-CoV 2 PCR or IgG test results	8 (13)	2 (29)
Participants with only negative SARS-CoV-2 PCR test results	28 (47)	2 (29)

Abbreviations: EDSS, Expanded Disability Status Scale; HSCT, hematopoietic stem cell transplantation; IgG, immunoglobulin G; IVIG, intravenous immunoglobulin; MS, multiple sclerosis; NA, not applicable; PCR, polymerase chain reaction; PPMS, primary progressive multiple sclerosis; RRMS, relapsing-remitting multiple sclerosis; SPMS, secondary progressive multiple sclerosis.

### Safety

The vaccination strategy resulted in a median (range) rituximab postponement of 11 (−3 to 37) weeks. Three patients (5%) had MS-associated disease activity between the last rituximab infusion before vaccination and the end of the study. One patient had a mild clinical relapse with numbness in the legs lasting less than 1 week. Two additional patients had subclinical magnetic resonance imaging–documented MS-associated disease activity. No unexpected adverse effects, severe MS-associated disease activity, or cases of COVID-19 infection were reported after vaccination.

### Initial B-Cell Counts and Kinetics of B-Cell Recovery

Approximately 6 months after the last rituximab infusion (median, 26 weeks; range, 25-34 weeks), data on B-cell counts were available for 48 patients. A wide diversity in the initial B-cell counts (median, 17/μL; range, 0/μL-163/μL) was observed (eFigure 2 in the Supplement). Nineteen patients (40%) had a B-cell count lower than 10/μL (median, 1/μL; range, 0/μL-8/μL), and 29 patients (60%) had a B-cell count of at least 10/μL (median, 37/μL; range, 11/μL-163/μL). In the group with B-cell counts lower than 10/μL, the rate of increase in B-cell counts was lower (simple linear regression line slope, 7.5; *R*^2^, 0.43, *P* < .001) than for patients with at least 10/μL (simple linear regression line slope, 18.2; *R*^2^, 0.24; *P* < .001), with a significant difference in the regression line slopes (*P* = .007).

To investigate factors associated with slow mobilization of B cells (B-cell counts <10/μL at 6 months), we created a logistic regression model in which age, sex, accumulated dose, treatment duration, and treatment interval were covariates and B-cell count lower than 10/μL or at least 10/μL as the binary classifier. Treatment duration was the only variable that had a nonoverlapping 95% CI in the model. Slow mobilizers had a median (range) treatment duration of 4.0 (0.8-6.9) years and fast mobilizers, 2.1 (0.5-6.0) years (*P* = .002).

### Humoral Immune Response

**Figure 1.**
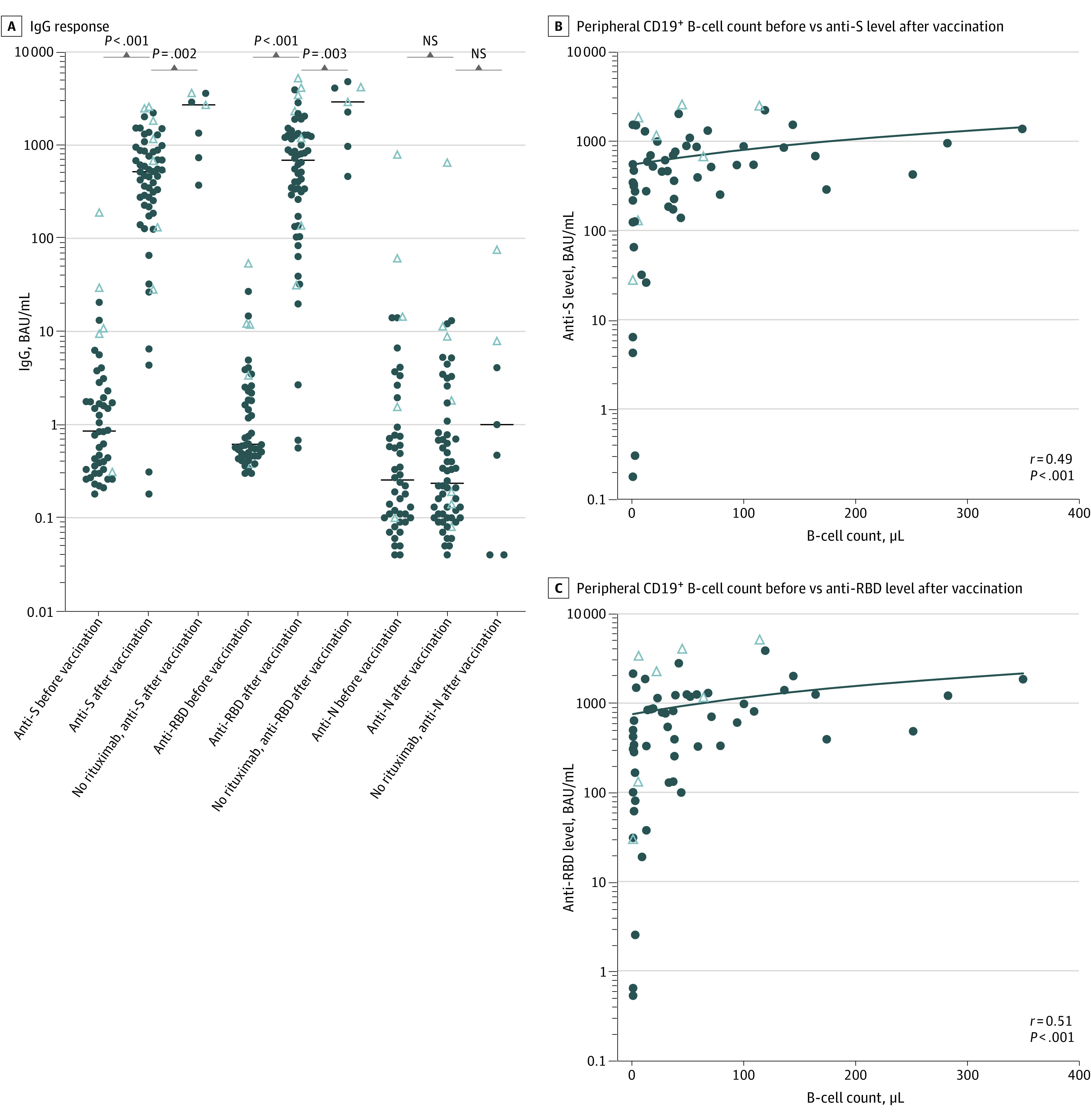
Humoral Immune Response to SARS-CoV-2 Proteins After Vaccination With Tozinameran A, Immunoglobulin G (IgG) response to spike (S), receptor-binding domain (RBD), and nucleocapsid proteins after SARS-CoV-2 vaccination in patients with multiple sclerosis who were receiving rituximab and in anti-CD20–naive patients with multiple sclerosis. Horizontal lines represent median values; closed circles, patients with negative test results for SARS-CoV-2 using a polymerase chain reaction (PCR) test prior to vaccination; open triangles, patients with a positive test result for SARS-CoV-2 using PCR test prior to vaccination; and NS, not statistically significant. B, Correlation between peripheral CD19^+^ B-cell count before vaccination and the level of anti-S IgG antibodies (anti-S) after vaccination. C, Correlation between peripheral CD19^+^ B-cell count before vaccination and the level of anti-RBD antibodies after vaccination. Anti-N represents antinucleocapsid IgG antibodies; BAU, binding antibody units.

**Figure 2.**
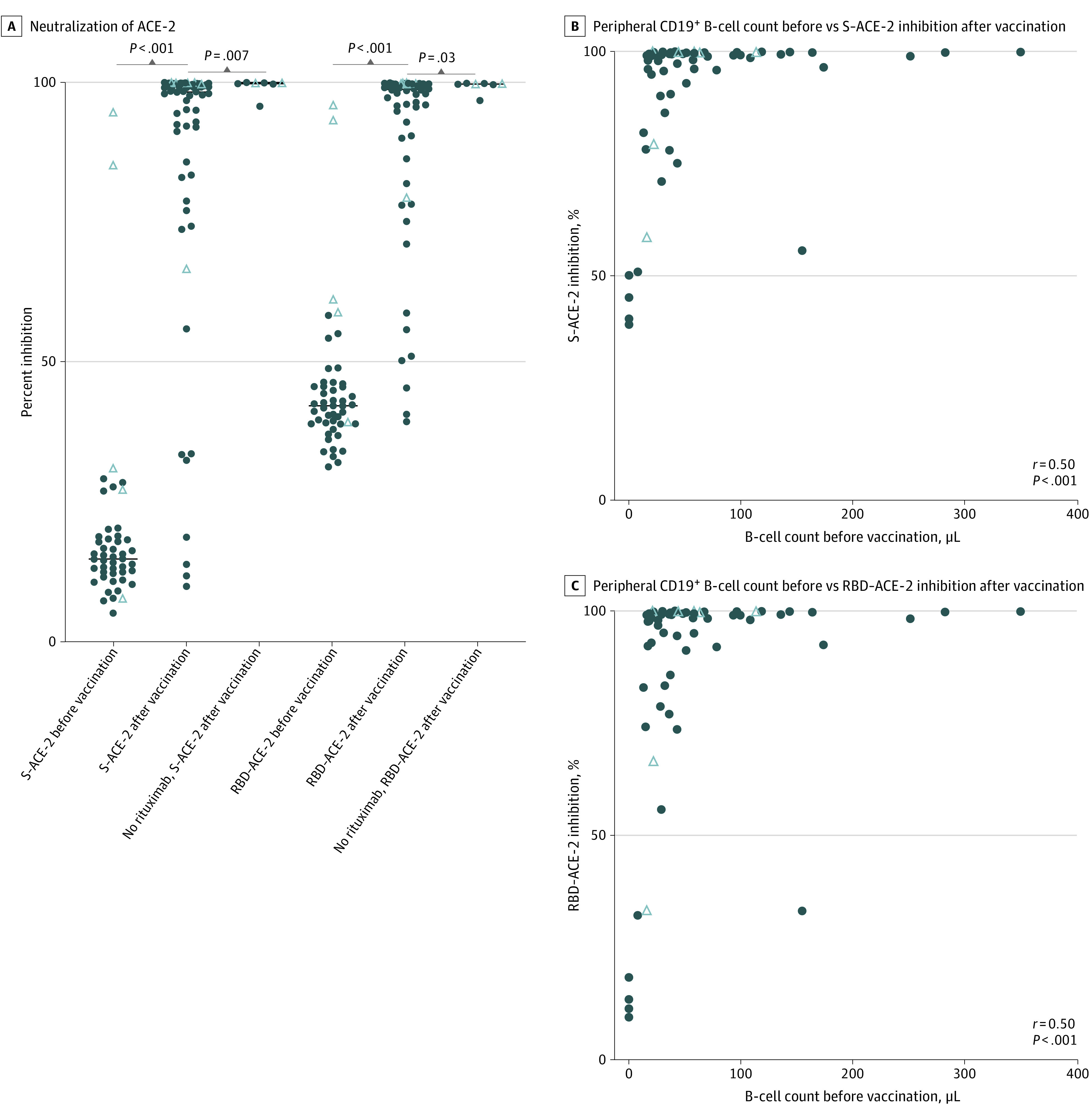
Angiotensin-Converting Enzyme 2 (ACE-2) Neutralizing Capacity of Anti-Spike (Anti-S) and Anti-Receptor-Binding Domain (Anti-RBD) Antibodies After Vaccination with Tozinameran A, Association of anti-S and anti-RBD antibodies with neutralization of ACE-2, measured as percent inhibition of S–ACE-2 and RBD–ACE-2 binding before and after SARS-CoV-2 vaccination in patients with multiple sclerosis receiving ongoing rituximab treatment and anti-CD20–naive patients with multiple sclerosis. Horizontal lines represent median values; closed circles, patients with negative test results for SARS-CoV-2 using a polymerase chain reaction (PCR) test prior to vaccination; and open triangles, patients with a positive test result for SARS-CoV-2 using PCR test prior to vaccination. B, Correlation between peripheral CD19^+^ B-cell count before vaccination and S–ACE-2 inhibition after vaccination. C, Correlation between peripheral CD19^+^ B-cell count before vaccination and RBD–ACE-2 inhibition after vaccination.

Serum samples from 48 patients before vaccination and samples from 60 patients after vaccination who were treated with rituximab were analyzed as well as 7 samples after vaccination from patients with MS who were never exposed to anti-CD20 treatment. The anti-S IgG antibodies were detected at protective levels (>264 BAU/mL) in 43 of 60 patients (72%) treated with rituximab after vaccination, and anti-RBD antibodies were detected at protective levels (>506 BAU/mL) in 34 of 60 patients (57%). However, 17 of 60 patients (28%) did not attain protective levels of anti-S or anti-RBD antibodies after vaccination. Anti-S and anti-RBD antibody levels did increase after vaccination in patients treated with rituximab, but the response was lower than in patients with MS without anti-CD20 treatment (Figure 1A). The anti-S and anti-RBD antibody levels were correlated with the B-cell counts in peripheral blood (Figure 1B and C). The median (range) B-cell count in rituximab-treated patients who attained neither protective anti-S nor anti-RBD antibody levels was 22/μL (0/μL-154/μL) compared with 51/μL (13/μL-348/μL) in the remainder of the cohort (*P* < .001). Of 60 rituximab-treated patients, 45 (75%) reached 90% or greater inhibition of S–ACE-2 binding, and 44 (73%) reached 90% or greater inhibition of RBD–ACE-2 binding. All 7 patients who were anti-CD20–naive reached 90% or greater inhibition of both S–ACE-2 and RBD–ACE-2 binding (Figure 2A). Inhibition of both S–ACE-2 and RBD–ACE-2 binding was correlated with B-cell counts in peripheral blood (Figure 2B and C). Inhibition of S–ACE-2 and RBD–ACE-2 was correlated with anti-S antibody levels and anti-RBD antibody levels, respectively (eFigure 4 in the Supplement).

### T-Cell–Mediated Immune Response

**Figure 3.**
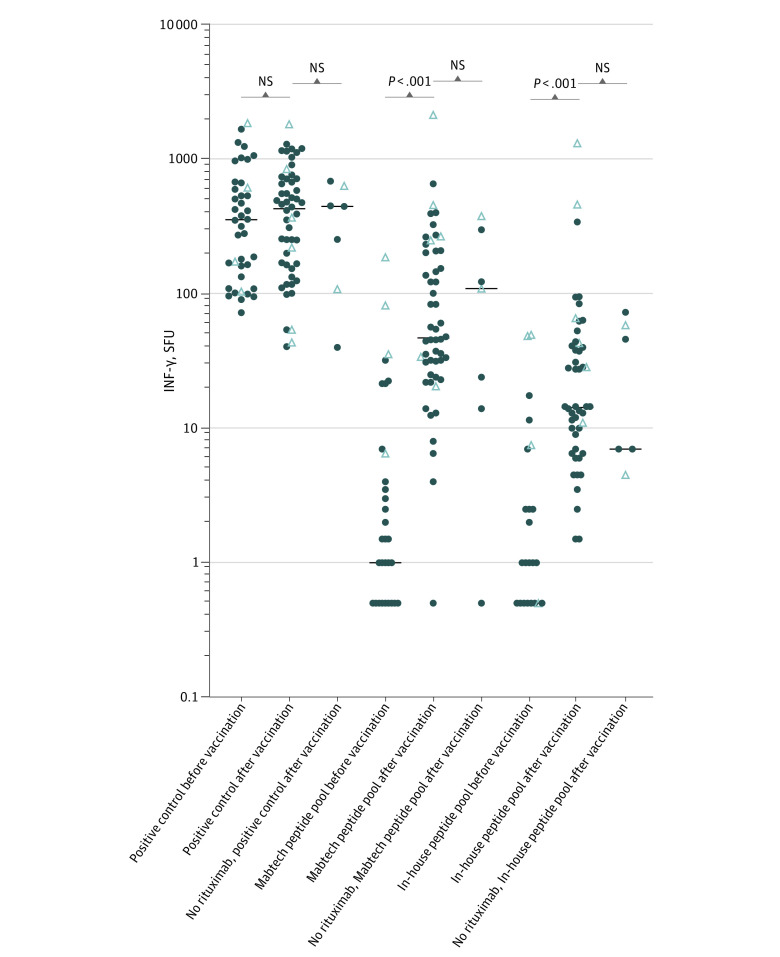
Cellular Response to SARS-CoV-2–Derived Peptides After Vaccination With Tozinameran Interferon gamma (IFN-γ) secretion in response to 24-hour stimulation with a positive control (anti-cluster of differentiation 3 antibodies), a commercially available peptide pool containing 100 peptides derived from the spike protein of SARS-CoV-2, or an in-house peptide pool containing 8 validated peptides from the spike protein of SARS-CoV-2. Secretion was quantitated by counting spot-forming units (SFUs) in a FluoroSpot assay. Horizontal lines represent median values; closed circles, patients with negative test results for SARS-CoV-2 using a polymerase chain reaction (PCR) test prior to vaccination; open triangles, patients with positive SARS-CoV-2 test results using PCR test prior to vaccination. NS indicates not statistically significant.

Peripheral blood mononuclear cells (PBMCs) obtained prior to vaccination from 40 patients with MS treated with rituximab and PBMCs obtained after vaccination from 52 patients were analyzed as well as PBMCs obtained after vaccination from 7 CD20-naive patients with MS. Stimulation with 2 different spike protein peptide pools resulted in increased IFN-γ SFUs in rituximab-treated patients after vaccination, with similar results in CD20-naive patients (Figure 3 and eFigures 6, 7, and 8 in the Supplement). An analysis of cells secreting interleukin 2 and cells co-secreting IFN-γ and interleukin 2 rendered comparable results (eFigure 5 in the Supplement). After vaccination, the IFN-γ SFU response was positive in 42 of 46 (91%) rituximab-treated patients with MS (with no history of a positive SARS-CoV-2 polymerase chain reaction test result) using the Mabtech peptide pool, and 38 of 46 patients (83%) using the in-house peptide pool. In 37 of 46 patients (80%), a positive IFN-γ response was observed with both peptide pools, and 3 of 46 patients (7%) were negative for an IFN-γ response with both pools.

### Factors Associated With Humoral and T-Cell Responses

In multiple linear regression analyses of the potential factors associated with the humoral and T-cell responses, only the B-cell count before vaccination was independently associated with anti-S IgG levels after vaccination (eTable 3 and eFigure 3 in the Supplement), and no variable was significantly associated with IFN-γ SFUs.

### B-Cell Count Cutoff

An analysis was conducted to determine the optimal cutoff of B-cell counts (Table 2 and eTable 4 in the Supplement). A cutoff value of 40/μL rendered a protective anti-S response in 26 of 29 rituximab-treated patients (90%), a protective anti-RBD response in 21 of 29 patients (72%), and an S–ACE-2 as well as an RBD–ACE-2 inhibition greater than 90% in 27 of 29 patients (93%). The T-cell mediated response was independent of B-cell count. At the B-cell cutoff of 40/μL, 22 of 25 patients (88%) had positive results in the T-cell assay using the Mabtech peptide pool, and 21 of 25 patients (84%) had positive results in the T-cell assay using the in-house peptide pool.

**Table 2.** Humoral and Cell-Mediated Response With Various Cutoff Values for B-Cell Count

Response	No. (%)					
	CD19^+^ B-cell count used as cutoff for vaccination, /μL					
	>0	>10	>20	>40	>60	>80
No. of patients	60	55	47	29	18	14
Antibody response, BAU/mL						
Patients with anti-S >264	43 (72)	43 (78)	38 (81)	26 (90)	16 (89)	13 (93)
Anti-S, mean (SD)	683 (634)	744 (627)	809 (650)	962 (709)	938 (664)	1008 (711)
Patients with anti-RBD>506	34 (57)	34 (62)	30 (64)	21 (72)	14 (78)	11 (79)
Anti-RBD, mean (SD)	988 (1102)	1077 (1109)	1186 (1158)	1457 (1307)	1445 (1297)	1601 (1426)
ACE-2 inhibition assay (% inhibition)						
Patients with ACE-2–S inhibition >90%	45 (75)	45 (82)	40 (85)	27 (93)	17 (94)	13 (93)
Inhibition ACE-2–S, mean (SD)	90 (17)	94 (10)	95 (9)	96 (9)	97 (10)	96 (12)
Patients with ACE-2–RBD inhibition >90%	44 (73)	44 (80)	39 (83)	27 (93)	17 (94)	13 (93)
Inhibition ACE-2–RBD, mean (SD)	86 (25)	92 (15)	93 (13)	95 (13)	95 (16)	94 (18)
Patients with positive T-cell response (%)						
No.	52	48	42	25	16	13
IFN-γ Mabtech peptide pool	48 (92)	44 (92)	38 (90)	22 (88)	13 (81)	11 (85)
IFN-γ in-house peptide pool	44 (85)	40 (83)	36 (86)	21 (84)	12 (75)	10 (77)

Abbreviations: ACE-2, angiotensin-converting enzyme 2; anti-RBD, anti–receptor-binding domain antibodies; anti-S, anti-spike antibodies; BAU, binding antibody units; IFN-γ, interferon gamma; S, spike.

## Discussion

A key finding of this cohort study was that the number of B cells in peripheral blood was associated with serological immune responses to vaccination with tozinameran in patients treated with the monoclonal anti-CD20 antibody rituximab.

Rituximab is a B-cell depleting agent that has increasingly been used off-label for treatment of MS in the last decade. It is well tolerated and associated with a large reduction in disease activity.^23,24^ In the early stages of the COVID-19 pandemic, it was reported that rituximab and other B-cell depleting agents were associated with an increased risk of developing severe COVID-19, which was consistent with results from larger studies^9^ and with a prepandemic study showing an increased risk of serious infections.^25^ Therapy with B-cell–depleting agents is also associated with a weakened or absent humoral vaccine response,^1,2,3,4,5,6^ which led to the recommendation that vaccinations should be made at the earliest 3 to 6 months after the last rituximab infusion.^10,14^ We surmised that time from last infusion was a proxy for B-cell counts, and we hypothesized that low B-cell counts would be associated with poor vaccine responses. When vaccines for SARS-CoV-2 became widely available, we therefore offered patients the opportunity to postpone their vaccination until their B-cell counts reached 20/μL (approximately 10% of the typical value for B-cell counts in peripheral blood) to increase their chances of getting a protective vaccine response. There were 2 main drawbacks with this strategy. If the effectiveness of the vaccination was not dependent on B-cell counts, patients would have been exposed to an unnecessary risk of COVID-19 that would have been prevented by an earlier vaccination. Patients were instructed to view themselves as individuals at increased risk for serious COVID-19 and follow the US Centers for Disease Control and Prevention guidelines.^26^ None of the participants developed severe COVID-19 requiring hospitalization. Postponed treatment with rituximab may also lead to a resurgence of disease MS-associated activity even though evidence suggests that rituximab infusions can be safely postponed to an interval of more than 1 year.^27^ Reassuringly, only 1 patient experienced a mild relapse, with complete remission within a week.

Our multivariate regression analysis indicated that B-cell counts were the most decisive factor for an adequate serological response. In previous studies, time from last infusion has often been put forward as an explanatory variable for the various responses to SARS-CoV-2 vaccination among patients treated with B-cell–depleting agents.^12,13,28^ This observation is hardly surprising because time from last infusion is a major determinant of the B-cell count. Notably, our data showed a large interindividual variability in the rate of B-cell recovery. Six months after the last rituximab infusion, 40% of patients had B-cell counts lower than 10/μL. This calls into question the general recommendation of performing vaccinations 3 to 6 months after the last infusion of a B-cell–depleting agent. Instead, relying on the measurement of B-cell counts appears to be the rational approach and is feasible in many hospital settings. We also noted that patients partitioned into 2 groups depending on their initial B-cell counts at 6 months. Patients with low B-cell counts (<10/μL) were slow mobilizers and increased their B-cell count by a mean of 7.5 cells per month. For patients with B-cell counts of at least 10/μL, the rate of recovery was much higher, with a mean of 18 cells per month. Using these data, it should be possible to make a rough estimate of when the target value of the B-cell count can be reached.

A previous showed that antibody levels of 264 BAU/mL for anti-S and 506 BAU/mL for anti-RBD were associated with 80% vaccine effectiveness against primary symptomatic COVID-19^20^; therefore, we used these values as targets for an adequate vaccine response. The proportion of patients reaching these target values increased with B-cell counts to a level of at least 40/μL, when the number of patients with a positive response started to approach its maximum value. One of the main functions of the anti-S and anti-RBD antibodies is to inhibit the ability of the SARS-CoV-2 virus to bind to ACE-2, which is the functional receptor on cell surfaces through which the virus enters host cells. Therefore, the neutralizing capacity of the antibodies should also be considered. Using a cutoff of 90% for the ACE-2 inhibition assay, 93% of patients developed functional neutralizing antibodies if their B-cell count was at least 40/μL. Some patients developed high levels of functional anti-S and anti-RBD antibodies despite low B-cell counts. Thus, if the priority lies with generating a vaccine response in *some* patients, vaccination should be considered early. But if a robust serological vaccine response in *all* patients is prioritized, then vaccination should be postponed until the B-cell count reaches at least 40/μL.

Analyzing T-cell responses to SARS-CoV-2 infection is less straightforward than serological responses, and different methods have been used in the past.^11,12,16,17^ We used 2 different peptide pools derived from the SARS-CoV-2 spike protein. The in-house pool was designed to include 8 peptides in total but with broad human leukocyte antigen coverage. The number of peptides may limit the sensitivity of the in-house pool but increases its specificity.^21^ The commercial pool does not provide sequence information but is sold as a SARS-CoV-2 S defined pool. The increased number of peptides in the commercial pool likely improves its sensitivity but may compromise its specificity owing to the known risk of sequence overlap between endemic coronaviruses and SARS-CoV-2.^22,29,30^ We used a FluoroSpot assay to evaluate cell-mediated immunity because of its superior sensitivity and its ability to simultaneously measure different analytes secreted at a single-cell level.^31^ Nearly all patients developed a T-cell response, which was independent of the B-cell counts in our regression model.

### Limitations

This study has limitations. Decreasing levels of neutralizing antibodies after vaccination are a concern, and treatment with rituximab may increase the rate of waning. We measured the response 6 weeks after the second dose of the vaccination, when vaccine responses are likely at their peak, but the duration of the response was not studied and is unknown. Booster doses of the vaccination may improve the longevity of vaccine responses, but this was also not investigated. Further studies are needed to address these issues. In previous studies, a robust T-cell response was associated with better outcome of COVID-19 infection,^32,33^ but established cutoff values connected to clinical efficacy are still lacking. It is therefore difficult to know to what degree the T-cell response protects against future infection with SARS-CoV-2 or whether it diminishes the severity of COVID-19 disease, especially in the face of a poor serological response.

## Conclusions

This cohort study found that the number of B cells in the peripheral blood was associated with the immune responses to vaccination with tozinameran among patients treated with the monoclonal anti-CD20 antibody rituximab. These results favor early vaccination without considering time from last infusion or B-cell count because some patients generated a functional serological response anyway, and T-cell responses appeared to develop independently of B-cell count. The results also suggest that an additional vaccine dose may be considered when the B-cell count reaches 40/μL to ensure that as many patients as possible will generate an adequate vaccine response. This approach is achievable by delaying treatment with rituximab, which appeared in the present study to be safe. It is possible that the approach used in this study may also be used to assess the feasibility of vaccination using other mRNA vaccines against SARS-CoV-2 as well as for patients treated with other monoclonal anti-CD20 antibodies. Further studies are needed to determine whether a similar approach is useful for other vaccines.

## Notes

Supplement.
eMethods.
**eFigure 1.** Flowchart Diagram of Patient Inclusion and Exclusion in the Study **eFigure 2.** B-Cell Recovery After Rituximab Infusion **eFigure 3.** Correlation Between Levels of Anti-S Antibodies or Anti-RBD Antibodies and CD4 T-Cell Count or CD8 T-Cell Count **eFigure 4.** The Ability to Neutralize ACE-2 for Anti-Spike Antibodies and Anti-RBD Antibodies Correlated Strongly to Anti-Spike and Anti-RBD IgG Antibody Levels, Respectively **eFigure 5.** Cell-Mediated Response Defined as Secretion of IL-2 and Co-Secretion of IL-2 + IFNγ Before and After Vaccination in Rituximab-Treated MS Patients as Well as in MS Patients Never Treated With Anti-CD20 **eFigure 6.** IFNγ Secretion in Patients With and Without Protective Antibody Levels of Anti-Spike or Anti-RBD, When Stimulated for 24 h With the In-House Peptide Pool (Eights) or Mabtech Peptide Pool (100S) **eFigure 7.** Correlation Between CD19+ B-Cell Count, CD4+ T-Cell Count, CD8+ T-Cell Count, and Cellular Immune Response Measured as IFNγ SFU After 24-h Stimulation With Mabtech Peptide Pool or an In-House Peptide Pool, After SARS-Cov-2 Vaccination **eFigure 8.** Correlation Between Levels of Anti-S Antibodies in Blood and Cellular Immune Response Measured as IFNγ SFU After 24-h Stimulation With the Mabtech Peptide Pool, or an In-House Peptide Pool After SARS-CoV-2 Vaccination **eTable 1.** Peptides Included in the In-House Generated SARS-CoV-2–Specific Peptide Pool **eTable 2.** Published HLA Types Covered by the SARS-CoV-2–Defined Peptide Pool Manufactured by Mabtech **eTable 3.** Results From Multiple Regression Analysis of Factors Affecting Anti-Spike Antibody Levels in Rituximab-Treated MS Patients After SARS-CoV-2 Vaccination **eTable 4.** Humoral and Cell-Mediated Response in Different Strata of B-Cell Counts

## References

[1] Oren S, Mandelboim M, Braun-Moscovici Y, . Vaccination against influenza in patients with rheumatoid arthritis: the effect of rituximab on the humoral response. Ann Rheum Dis. 2008;67(7):937-941. doi:10.1136/ard.2007.077461 17981914

[2] Bingham CO III, Looney RJ, Deodhar A, . Immunization responses in rheumatoid arthritis patients treated with rituximab: results from a controlled clinical trial. Arthritis Rheum. 2010;62(1):64-74. doi:10.1002/art.25034 20039397

[3] van Assen S, Holvast A, Benne CA, . Humoral responses after influenza vaccination are severely reduced in patients with rheumatoid arthritis treated with rituximab. Arthritis Rheum. 2010;62(1):75-81. doi:10.1002/art.25033 20039396

[4] Eisenberg RA, Jawad AF, Boyer J, . Rituximab-treated patients have a poor response to influenza vaccination. J Clin Immunol. 2013;33(2):388-396. doi:10.1007/s10875-012-9813-x 23064976 PMC3565069

[5] Nazi I, Kelton JG, Larché M, . The effect of rituximab on vaccine responses in patients with immune thrombocytopenia. Blood. 2013;122(11):1946-1953. doi:10.1182/blood-2013-04-494096 23851398 PMC3773242

[6] Bar-Or A, Calkwood JC, Chognot C, . Effect of ocrelizumab on vaccine responses in patients with multiple sclerosis: the VELOCE study. Neurology. 2020;95(14):e1999-e2008. doi:10.1212/WNL.0000000000010380 32727835 PMC7843152

[7] Houot R, Levy R, Cartron G, Armand P. Could anti-CD20 therapy jeopardise the efficacy of a SARS-CoV-2 vaccine? Eur J Cancer. 2020;136:4-6. doi:10.1016/j.ejca.2020.06.017 32619884 PMC7315961

[8] Sormani MP, De Rossi N, Schiavetti I, ; Musc-19 Study Group. Disease-modifying therapies and coronavirus disease 2019 severity in multiple sclerosis. Ann Neurol. 2021;89(4):780-789. doi:10.1002/ana.26028 33480077 PMC8013440

[9] Spelman T, Forsberg L, McKay K, Glaser A, Hillert J. Increased rate of hospitalisation for COVID-19 among rituximab-treated multiple sclerosis patients: a study of the Swedish multiple sclerosis registry. Published online ahead of print. Mult Scler. 2021;13524585211026272. doi:10.1177/13524585211026272 34212816

[10] Disanto G, Sacco R, Bernasconi E, . Association of disease-modifying treatment and anti-cd20 infusion timing with humoral response to 2 SARS-CoV-2 vaccines in patients with multiple sclerosis. JAMA Neurol. 2021;78(12):1529-1531. doi:10.1001/jamaneurol.2021.3609 34554185 PMC8461551

[11] Apostolidis SA, Kakara M, Painter MM, . Cellular and humoral immune responses following SARS-CoV-2 mRNA vaccination in patients with multiple sclerosis on anti-CD20 therapy. Nat Med. 2021;27(11):1990-2001. doi:10.1038/s41591-021-01507-2 34522051 PMC8604727

[12] Brill L, Rechtman A, Zveik O, . Humoral and T-cell response to SARS-CoV-2 vaccination in patients with multiple sclerosis treated with ocrelizumab. JAMA Neurol. 2021;78(12):1510-1514. doi:10.1001/jamaneurol.2021.3599 34554197 PMC8461553

[13] König M, Lorentzen AR, Torgauten HM, . Humoral immunity to SARS-CoV-2 mRNA vaccination in multiple sclerosis: the relevance of time since last rituximab infusion and first experience from sporadic revaccinations. J Neurol Neurosurg Psychiatry. 2021;jnnp-2021-327612. doi:10.1136/jnnp-2021-327612 34670844 PMC9763174

[14] Arnold J, Winthrop K, Emery P. COVID-19 vaccination and antirheumatic therapy. Rheumatology (Oxford). 2021;60(8):3496-3502. doi:10.1093/rheumatology/keab223 33710296 PMC7989162

[15] The Swedish MS Association. Checklista inför vaccination under pågående MS-behandling [in Swedish]. Published January 20, 2020. Accessed December 1, 2021. https://www.mssallskapet.se/wp-content/uploads/2020/02/checklista-inför-vaccination-under-pågående-MS-behandling-1.pdf

[16] Madelon N, Lauper K, Breville G, . Robust T cell responses in anti-CD20 treated patients following COVID-19 vaccination: a prospective cohort study. Clin Infect Dis. 2021;ciab954. doi:10.1093/cid/ciab954 34791081 PMC8767893

[17] Pompsch M, Fisenkci N, Horn PA, Kraemer M, Lindemann M. Evidence of extensive cellular immune response after SARS-CoV-2 vaccination in ocrelizumab-treated patients with multiple sclerosis. Neurol Res Pract. 2021;3(1):60. doi:10.1186/s42466-021-00158-5 34802469 PMC8606235

[18] UK EQATOR Centre. The Strengthening the Reporting of Observational Studies in Epidemiology (STROBE) Statement: guidelines for reporting observational studies. Accessed March 20, 2022. https://www.equator-network.org/reporting-guidelines/strobe/10.1136/bmj.39335.541782.AD PMC2034723 17947786

[19] World Medical Association. World Medical Association Declaration of Helsinki: ethical principles for medical research involving human subjects. JAMA. 2013;310(20):2191-2194. doi:10.1001/jama.2013.281053 24141714

[20] Feng S, Phillips DJ, White T, ; Oxford COVID Vaccine Trial Group. Correlates of protection against symptomatic and asymptomatic SARS-CoV-2 infection. Nat Med. 2021;27(11):2032-2040. doi:10.1038/s41591-021-01540-1 34588689 PMC8604724

[21] Mangsbo SM, Havervall S, Laurén I, . An evaluation of a FluoroSpot assay as a diagnostic tool to determine SARS-CoV-2 specific T cell responses. PLoS One. 2021;16(9):e0258041. doi:10.1371/journal.pone.0258041 34591918 PMC8483319

[22] Havervall S, Ng H, Jernbom Falk A, . Robust humoral and cellular immune responses and low risk for reinfection at least 8 months following asymptomatic to mild COVID-19. J Intern Med. 2022;291(1):72-80. doi:10.1111/joim.13387 34459525 PMC8661920

[23] Salzer J, Svenningsson R, Alping P, . Rituximab in multiple sclerosis: A retrospective observational study on safety and efficacy. Neurology. 2016;87(20):2074-2081. doi:10.1212/WNL.0000000000003331 27760868 PMC5109942

[24] Hauser SL, Waubant E, Arnold DL, ; HERMES Trial Group. B-cell depletion with rituximab in relapsing-remitting multiple sclerosis. N Engl J Med. 2008;358(7):676-688. doi:10.1056/NEJMoa0706383 18272891

[25] Luna G, Alping P, Burman J, . infection risks among patients with Multiple Sclerosis treated with fingolimod, natalizumab, rituximab, and injectable therapies. JAMA Neurol. 2020;77(2):184-191. doi:10.1001/jamaneurol.2019.3365 31589278 PMC6784753

[26] Centers for Disease Control and Prevention. How to protect yourself & others. Updated February 25, 2022. Accessed December 1, 2021. https://www.cdc.gov/coronavirus/2019-ncov/prevent-getting-sick/prevention.html

[27] Juto A, Fink K, Al Nimer F, Piehl F. Interrupting rituximab treatment in relapsing-remitting multiple sclerosis; no evidence of rebound disease activity. Mult Scler Relat Disord. 2020;37:101468. doi:10.1016/j.msard.2019.101468 31683231

[28] Sormani MP, Inglese M, Schiavetti I, ; CovaXiMS study group on behalf of the Italian Covid-19 Alliance in MS. Effect of SARS-CoV-2 mRNA vaccination in MS patients treated with disease modifying therapies. EBioMedicine. 2021;72:103581. doi:10.1016/j.ebiom.2021.103581 34563483 PMC8456129

[29] Mateus J, Grifoni A, Tarke A, . Selective and cross-reactive SARS-CoV-2 T cell epitopes in unexposed humans. Science. 2020;370(6512):89-94. doi:10.1126/science.abd3871 32753554 PMC7574914

[30] Le Bert N, Tan AT, Kunasegaran K, . SARS-CoV-2-specific T cell immunity in cases of COVID-19 and SARS, and uninfected controls. Nature. 2020;584(7821):457-462. doi:10.1038/s41586-020-2550-z 32668444

[31] Chauvat A, Benhamouda N, Gey A, . Clinical validation of IFNγ/IL-10 and IFNγ/IL-2 FluoroSpot assays for the detection of Tr1 T cells and influenza vaccine monitoring in humans. Hum Vaccin Immunother. 2014;10(1):104-113. doi:10.4161/hv.26593 24084262 PMC4181035

[32] Sekine T, Perez-Potti A, Rivera-Ballesteros O, ; Karolinska COVID-19 Study Group. Robust T Cell Immunity in Convalescent Individuals with Asymptomatic or Mild COVID-19. Cell. 2020;183(1):158-168.e14. doi:10.1016/j.cell.2020.08.017 32979941 PMC7427556

[33] Rydyznski Moderbacher C, Ramirez SI, Dan JM, . Antigen-specific adaptive immunity to SARS-CoV-2 in acute COVID-19 and associations with age and disease severity. Cell. 2020;183(4):996-1012.e19. doi:10.1016/j.cell.2020.09.038 33010815 PMC7494270

