# Supplementary material for: Factors Associated With Serological Response to SARS-CoV-2 Vaccination in Patients With Multiple Sclerosis Treated With Rituximab
Source: JAMA Netw Open. 2022 May 11;5(5):e2211497. doi: 10.1001/jamanetworkopen.2022.11497 (PMC9096596; doi:10.1001/jamanetworkopen.2022.11497)
Supplement: Supplement. — eMethods. eFigure 1. Flowchart Diagram of Patient Inclusion and Exclusion in the Study eFigure 2. B-Cell Recovery After Rituximab Infusion eFigure 3. Correlation Between Levels of Anti-S Antibodies or Anti-RBD Antibodies and CD4 T-Cell Count or CD8 T-Cell Count eFigure 4. The Ability to Neutralize ACE-2 for Anti-Spike Antibodies and Anti-RBD Antibodies Correlated Strongly to Anti-Spike and Anti-RBD IgG Antibody Levels, Respectively eFigure 5. Cell-Mediated Response Defined as Secretion of IL-2 and Co-Secretion of IL-2 + IFNγ Before and After Vaccination in Rituximab-Treated MS Patients as Well as in MS Patients Never Treated With Anti-CD20 eFigure 6. IFNγ Secretion in Patients With and Without Protective Antibody Levels of Anti-Spike or Anti-RBD, When Stimulated for 24 h With the In-House Peptide Pool (Eights) or Mabtech Peptide Pool (100S) eFigure 7. Correlation Between CD19+ B-Cell Count, CD4+ T-Cell Count, CD8+ T-Cell Count, and Cellular Immune Response Measured as IFNγ SFU After 24-h Stimulation With Mabtech Peptide Pool or an In-House Peptide Pool, After SARS-Cov-2 Vaccination eFigure 8. Correlation Between Levels of Anti-S Antibodies in Blood and Cellular Immune Response Measured as IFNγ SFU After 24-h Stimulation With the Mabtech Peptide Pool, or an In-House Peptide Pool After SARS-CoV-2 Vaccination eTable 1. Peptides Included in the In-House Generated SARS-CoV-2–Specific Peptide Pool eTable 2. Published HLA Types Covered by the SARS-CoV-2–Defined Peptide Pool Manufactured by Mabtech eTable 3. Results From Multiple Regression Analysis of Factors Affecting Anti-Spike Antibody Levels in Rituximab-Treated MS Patients After SARS-CoV-2 Vaccination eTable 4. Humoral and Cell-Mediated Response in Different Strata of B-Cell Counts [file jamanetwopen-e2211497-s001.pdf]

Supplementary Online Content

Tolf A, Wiberg A, Müller M, et al. Factors associated with serologic response to SARS-CoV-2 vaccination in patients with multiple sclerosis treated with rituximab. *JAMA Netw Open*. 2022;5(5):e2211497. doi:10.1001/jamanetworkopen.2022.11497

eMethods.
eFigure 1. Flowchart diagram of Patient Inclusion and Exclusion in the Study
eFigure 2. B-Cell Recovery After Rituximab Infusion
eFigure 3. Correlation Between Levels of Anti-S Antibodies or Anti-RBD Antibodies and CD4 T-Cell Count or CD8 T-Cell Count
eFigure 4. The Ability To Neutralize ACE-2 for Anti-Spike Antibodies and Anti-RBD Antibodies Correlated Strongly to Anti-S and Anti-RBD IgG Antibody Levels, Respectively
eFigure 5. Cell-Mediated Response Defined as Secretion of IL-2 and Co-Secretion of IL-2 + IFNγ Before and After Vaccination in Rituximab-Treated Patients With MS as Well as in Patients With MS Never Treated With Anti-CD20
eFigure 6. IFNγ Secretion in Patients With and Without Protective Antibody Levels of Anti-Spike or Anti-RBD, When Stimulated for 24 h With the In-House Peptide Pool (Eights) or Mabtech Peptide Pool (100S)
eFigure 7. Correlation Between CD19+ B-Cell Count, CD4+ T Cell Count, CD8+ T Cell Count, and Cellular Immune Response Measured as IFNγ SFU After 24-h Stimulation With Mabtech Peptide Pool or an In-House Peptide Pool, After SARS-Cov-2 Vaccination
eFigure 8. Correlation Between Levels of Anti-S Antibodies in Blood and Cellular Immune Response Measured as IFNγ SFU After 24-h Stimulation With the Mabtech Peptide Pool, or an In-House Peptide Pool After SARS-CoV-2 Vaccination
eTable 1. Peptides Included in the In-House Generated SARS-CoV-2–Specific Peptide Pool
eTable 2. Published HLA Types Covered by the SARS-CoV-2–Defined Peptide Pool Manufactured by Mabtech
eTable 3. Results From Multiple Regression Analysis of Factors Affecting Anti-Spike Antibody Levels in Rituximab-Treated Patients With MS After SARS-CoV-2 Vaccination
eTable 4. Humoral and Cell-Mediated Response in Different Strata of B-Cell Counts

This supplementary material has been provided by the authors to give readers additional information about their work.

© 2022 Tolf A et al. *JAMA Network Open*.

eMethods.

Evaluation of cell-mediated response after SARS-CoV-2 vaccination

SARS-CoV-2 specific T-cell reactivity was determined using a human IFNγ IL-2 FluoroSpot assay (Mabtech, Sweden). Peripheral blood mononuclear cells (PBMCs) were rested overnight prior to stimulation in complete RPMI 1640 with GlutaMaxTM medium (Gibco), supplemented with 10% FBS (Gibco) and 100 units of penicillin-streptomycin (Gibco). Cells were then stimulated for 24h at 37° C with 5% CO_2 with either a commercial peptide pool from Mabtech containing 100 peptides derived from the human SARS-CoV-2 spike protein (product 3630-1, SARS-CoV-2 S defined peptide pool) or a previously characterized peptide selection (8 peptides listed in Supplementary table 1) derived from the spike protein, (EightS).[18, 19] Anti-CD3 (CD3-2, Mabtech) was used as a positive control and DMSO was used as a negative control. Cells were plated in duplicates with $2{,}5x10^5$ cells/well, and $0{,}5x10^5$ cells/well in the positive anti-CD3 control. SARS-CoV-2 specific peptide pools were added at a concentration of 2 µg/ml for each individual peptide. Following development of the plates using the kit reagents, spot counts were read using a Mabtech IRIS and spots were analyzed using Mabtech Apex software 1.1. Mean spot counts in DMSO-treated negative control wells were deducted from the means to generate normalized spot counts for all other treated wells.

Additional information on the use of statistical methods

Summary characteristics were described using means for normally distributed variables and otherwise as medians. Most of the variables were not normally distributed and therefore the Wilcoxon matched-pairs signed rank tests and Mann-Whitney tests were used for comparisons between groups. Spearman's rank coefficient of correlation was used to describe correlations (Figure 2B, C, E, F; Supplementary figure 2, 3, 6 and 7).

Comparisons between pre- and post-vaccination samples (Figure 2A, D; Figure 3; Supplementary figure 4) were carried out using Wilcoxon matched-pairs signed rank test, as these were paired samples. The Mann-Whitney test was used when independent samples were compared. Specifically, for comparisons between slow mobilizers and fast mobilizers (Supplementary figure 1), comparisons between rituximab treated patients who did not attain protective anti-S or anti-RBD levels (Supplementary figure 5), comparisons between post-vaccination samples from patients treated with rituximab and post-vaccination samples from patients not treated with rituximab (Figure 2A, D; Figure 3; Supplementary figure 4).

A multiple logistic regression model was used to investigate which factors affected the dichotomous variables “slow mobilizer” and “fast mobilizer” in B-cell recovery. Multiple linear regression models were used to investigate which factors affected the continuous variables anti-spike IgG levels and IFNγ SFU levels.

© 2022 Tolf A et al. *JAMA Network Open*.

Supplementary figures

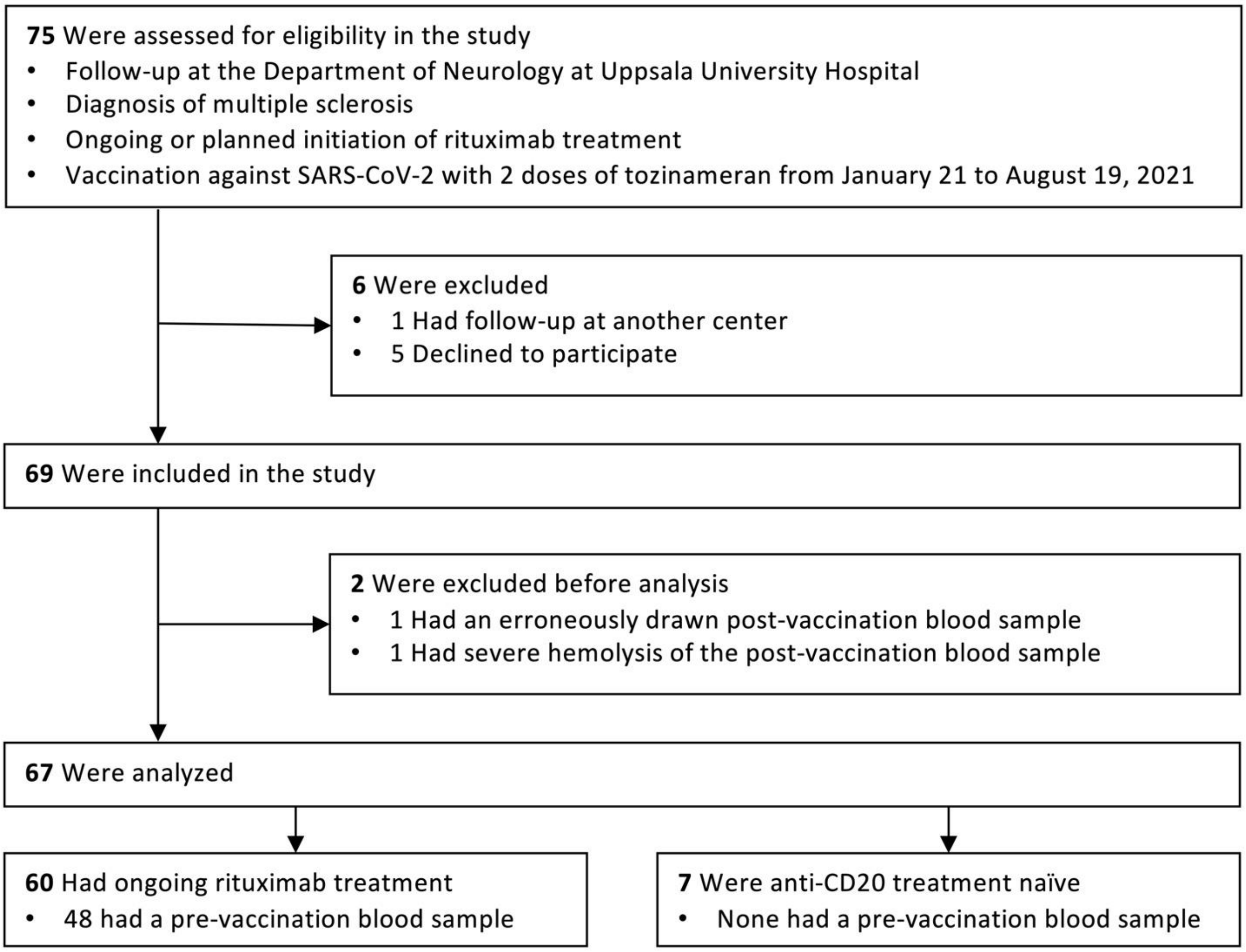

eFigure 1. Flowchart diagram of Patient Inclusion and Exclusion in the Study

© 2022 Tolf A et al. *JAMA Network Open*.

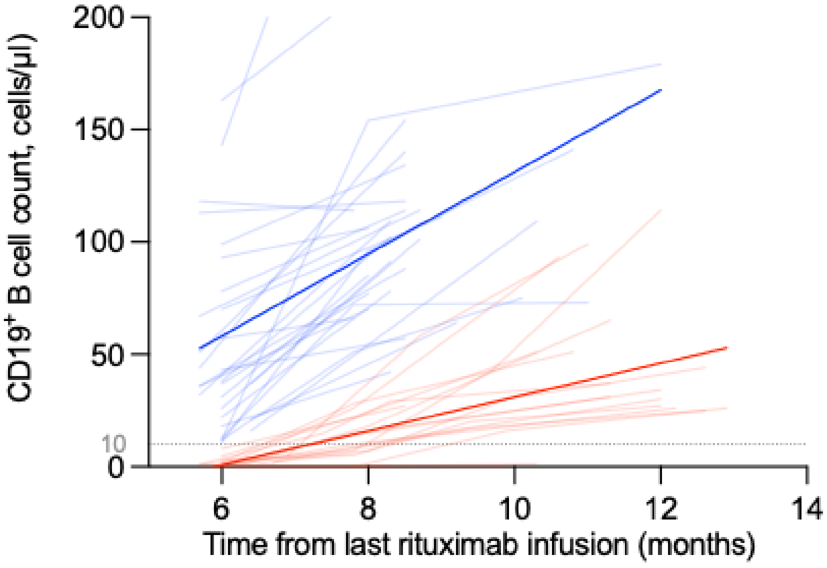

eFigure 2. B-Cell Recovery After Rituximab Infusion

The kinetics of B-cell recovery after rituximab treatment were different in patients depending on whether their B-cell count was <10 cells/µL or ≥10 cells/µL approximately 6 months after their last rituximab infusion. Patients with an initial B-cell count <10 cells/µL (n=19, shown in red) increased their B-cell count by on average 7.5 cells/µL/month, whereas patients with an initial B-cell count ≥10 cells/µL (n=29, shown in blue) increased their B-cell counts more rapidly, on average 18 B-cells/µL/month.

© 2022 Tolf A et al. *JAMA Network Open*.

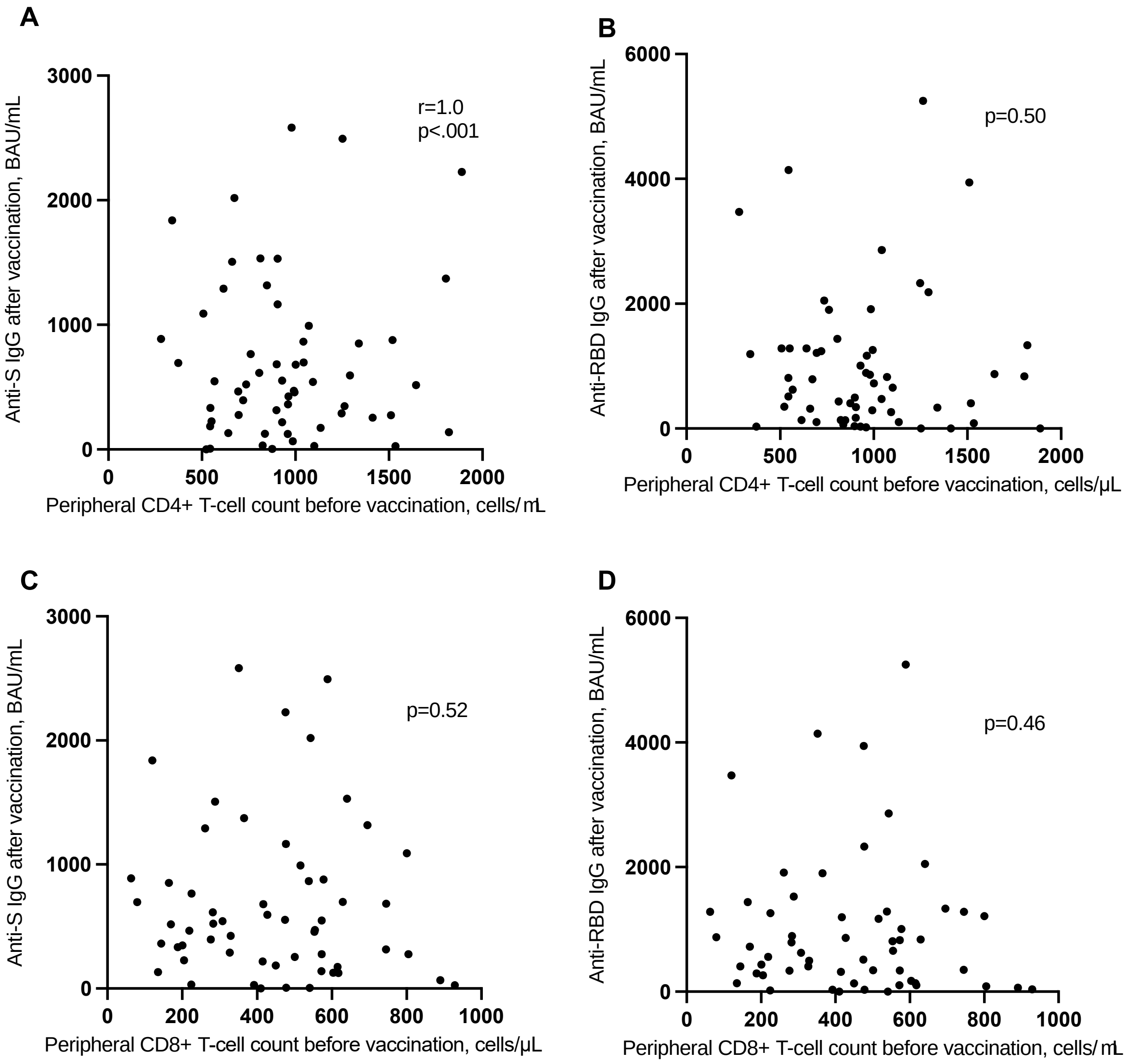

eFigure 3. Correlation Between Levels of Anti-S Antibodies (**A, C**) or Anti-RBD Antibodies (**B, D**) and CD4 T-Cell Count (**A, B**) or CD8 T-Cell Count (**C, D**). CD, cluster of differentiation; IgG, Immunoglobulin G; S, spike; RBD, receptor-binding domaine.

© 2022 Tolf A et al. *JAMA Network Open*.

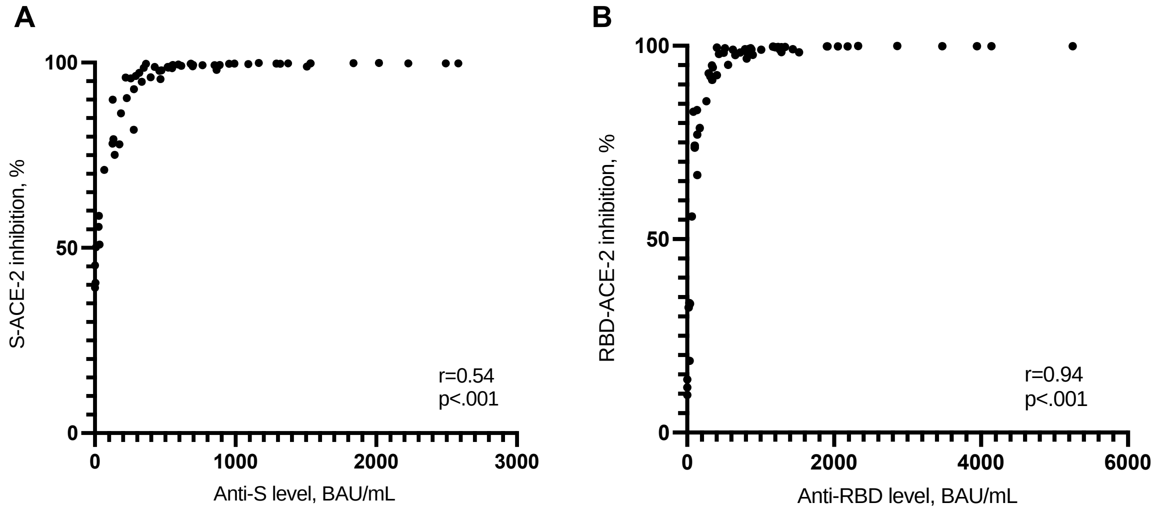

eFigure 4. The Ability To Neutralize ACE-2 for Anti-Spike Antibodies (**A**) and Anti-RBD Antibodies (**B**) Correlated Strongly to Anti-Spike and Anti-RBD Immunoglobulin G (IgG) Antibody Levels, Respectively
RBD, receptor binding domain; S, spike; ACE-2, angiotensin converting enzyme 2; BAU, Binding Antibody Units.

© 2022 Tolf A et al. *JAMA Network Open*.

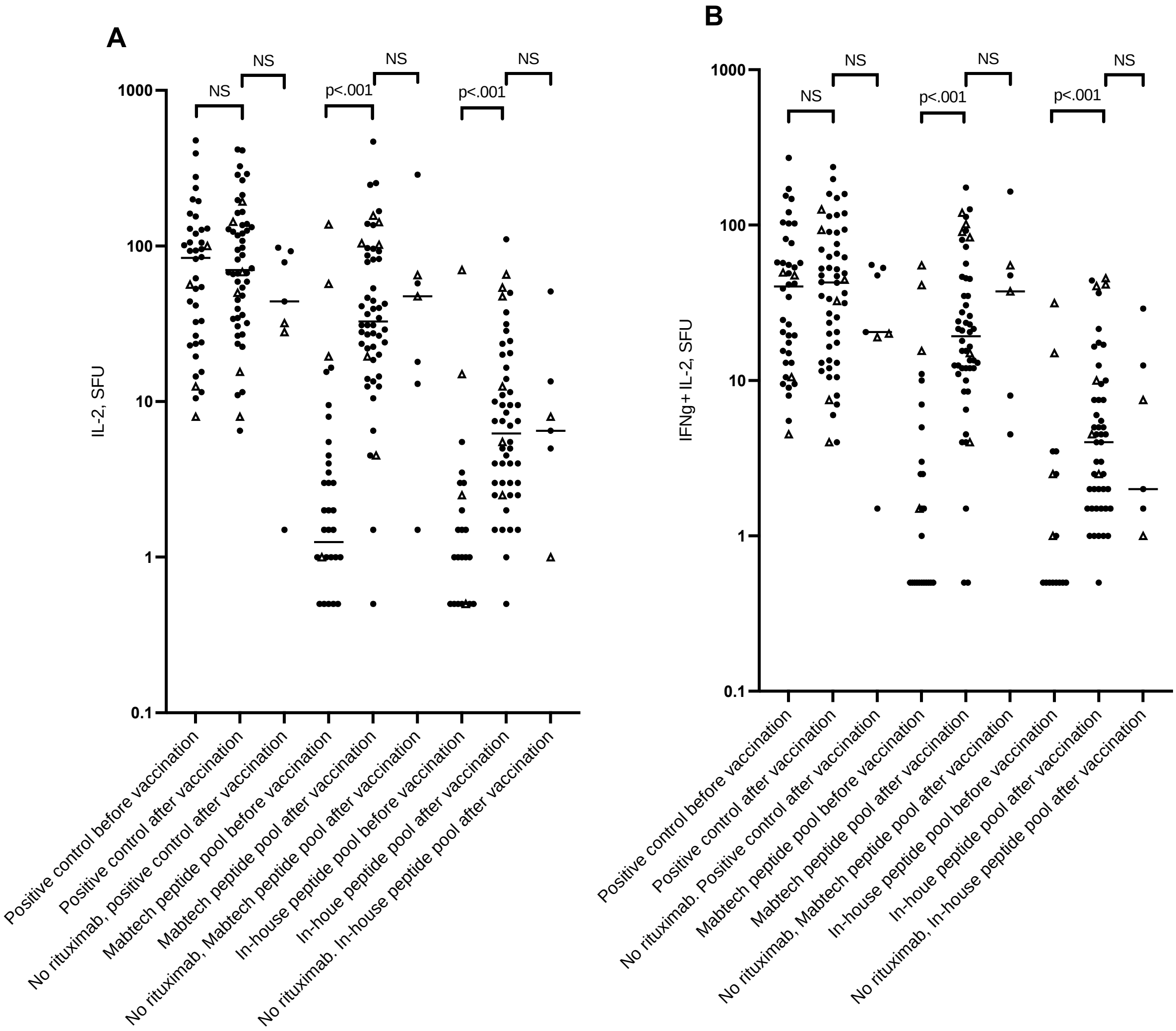

eFigure 5. Cell-Mediated Response Defined as Secretion of IL-2 (**A**) and Co-Secretion of IL-2 + IFNγ (**B**) Before and After Vaccination in Rituximab-Treated Patients With MS as Well as in Patients With MS Never Treated With Anti-CD20.

Lines mark median values. Open triangles, patients who had tested positive for SARS-CoV-2 with PCR previous to vaccination and pre-vaccination sampling; closed circles, patients with only negative test results for SARS-CoV-2 using polymerase chain reaction prior to vaccination; SFU, spot forming units; IFN, interferon; IL, interleukin; NS, not statistically significant.

© 2022 Tolf A et al. *JAMA Network Open*.

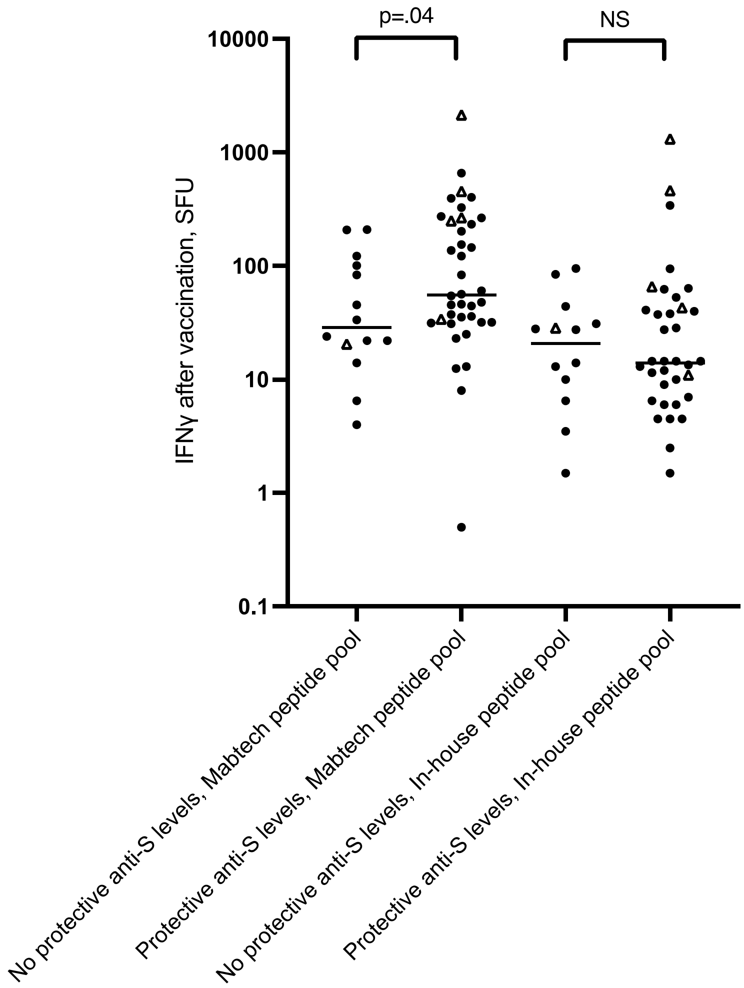

eFigure 6. IFNγ Secretion in Patients With and Without Protective Antibody Levels of Anti-Spike, When Stimulated for 24 h With the In-House Peptide Pool (EightS) or Mabtech Peptide Pool (100S).

Protective anti-S levels are defined as >264 BAU/ml. Lines mark median values. Open triangles, patients who had tested positive for SARS-CoV-2 with PCR previous to vaccination and pre-vaccination sampling; closed circles, patients with only negative test results for SARS-CoV-2 using polymerase chain reaction prior to vaccination; SFU, spot forming units; IFN, interferon; NS, not statistically significant.

© 2022 Tolf A et al. *JAMA Network Open*.

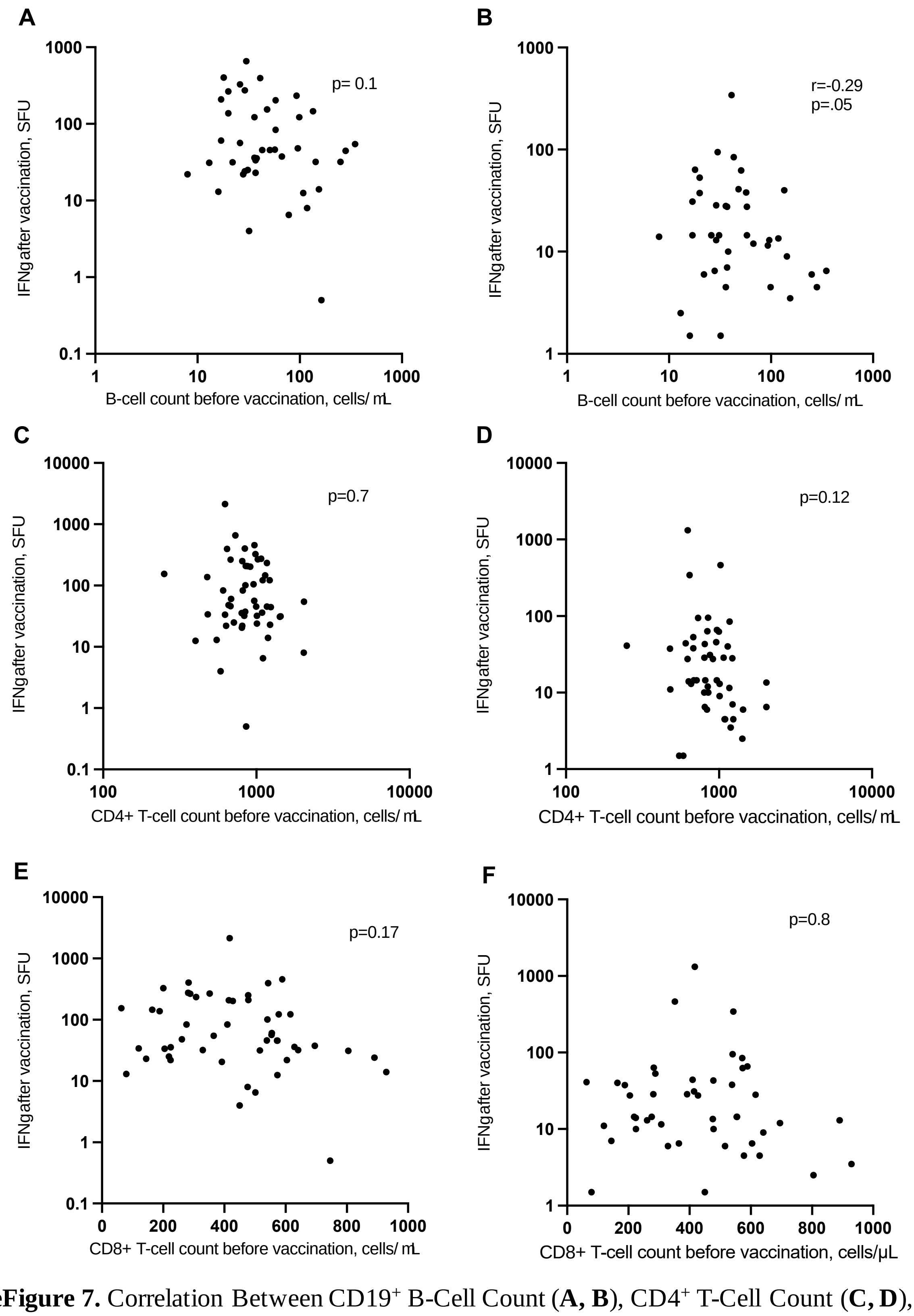

eFigure 7. Correlation Between CD19+ B-Cell Count (**A, B**), CD4+ T-Cell Count (**C, D**), CD8+ T-Cell Count (**E, F**) and Cellular Immune Response Measured as IFNγ SFU After 24-h Stimulation With Mabtech Peptide Pool (**A, C, E**) or an In-House Peptide Pool (**B, D, F**), After SARS-Cov-2 Vaccination

SFU, spot forming units; IFN, interferon; CD, cluster of differentiation.

© 2022 Tolf A et al. *JAMA Network Open.*

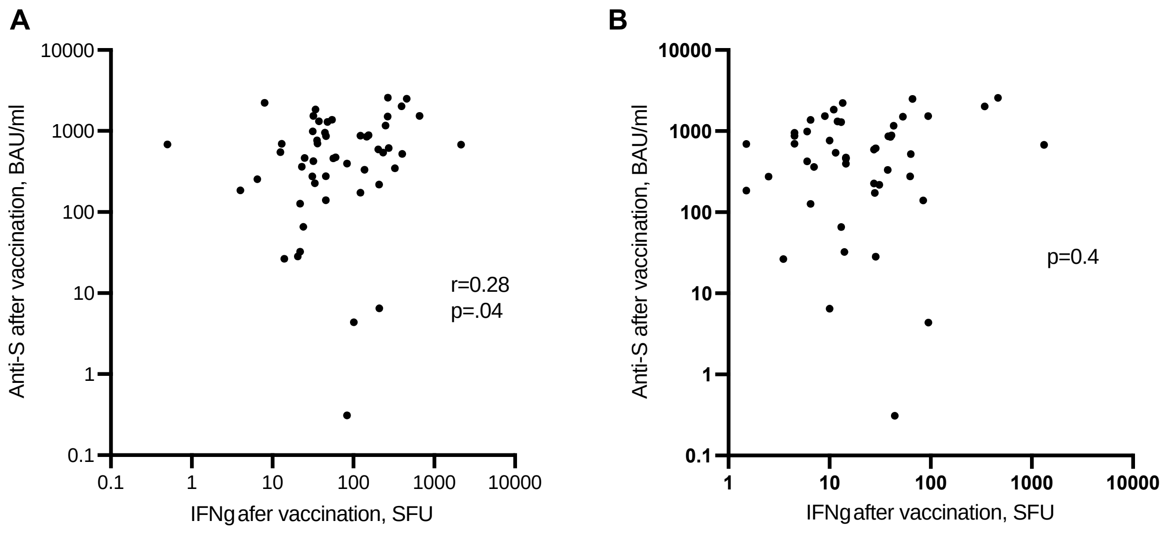

eFigure 8. Correlation Between Levels of Anti-S Antibodies in Blood and Cellular Immune Response Measured as IFNγ SFU After 24-h Stimulation With **A)** the Mabtech Peptide Pool, or **B)** an In-House Peptide Pool (EightS) After SARS-CoV-2 Vaccination

SFU, spot forming units; IFN, interferon; BAU, Binding Antibody Units.

© 2022 Tolf A et al. *JAMA Network Open*.

Supplementary tables

Protein	Position	Sequence
Spike	166-180	CTFEYVSQPFLMDLE
	261-285	GAAAYYVGYLQPRTFLLKYNENGTI
	365-394	YSVLYNSASFSTFKCYGVSPTKLNDLCFTN
	450-469	NYLYRLFRKSNLKPFERDIS
	495-514	YGFQPTNGVGYQPYRVVVLS
	562-586	FQQFGRDIADTTDAVRDPQTLEILD
	606-622	NQVAVLYQ**G**VNCTEVPV
	606-622	NQVAVLYQ**D**VNCTEVPV

eTable 1. Peptides Included in the In-House Generated SARS-CoV-2–Specific Peptide Pool

© 2022 Tolf A et al. *JAMA Network Open*.

A*01:01
A*02:01
A*03:01
A*11:01
A*24:02
A*26:01
A*29:02
A*30:02
A*31:01
A*68:01
B*07:02
B*08:01
B*15:01
B*35:01
B*40:01
B*44:02
B*44:03
C*07:01
DRB1*01:01
DRB1*03:01
DRB1*04:01
DRB1*04:04
DRB1*04:05
DRB1*07:01
DRB1*08:02
DRB1*09:01
DRB1*12:01
DRB1*13:01
DRB1*14:01
DRB1*15:01
DRB1*16:01
DRB3*01:01
DRB4*01:01
DQB1*02:01
DQB1*02:02
DQB1*03:01
DQB1*03:03
DQB1*04:02
DQB1*05:02
DQB1*05:03
DQB1*06:02
DQB1*06:03

eTable 2. Published HLA Types Covered by the SARS-CoV-2–Defined Peptide Pool Manufactured by Mabtech

© 2022 Tolf A et al. *JAMA Network Open*.

Parameter estimates	Variable	Estimate	Standard error	95% CI (asymptotic)	|t|	P value	P value summary
β0	Intercept	6.558	2.957	0.5901 to 12.53	2.218	0.0320	*
β1	Sex[M]	0.2018	0.1666	-0.1344 to 0.5381	1.211	0.2325	ns
β2	Age at vaccination	-0.4682	0.6235	-1.727 to 0.7901	0.7509	0.4569	ns
β3	No of previous anti-CD20 infusions	-0.07866	0.7404	-1.573 to 1.416	0.1062	0.9159	ns
β4	Accumulated dosage RTX	-0.4896	0.7916	-2.087 to 1.108	0.6185	0.5396	ns
β5	Positive PCR[J]	0.2433	0.1733	-0.1064 to 0.5931	1.404	0.1676	ns
β6	Time since anti-CD20-mAb (weeks)	0.06896	0.5480	-1.037 to 1.175	0.1258	0.9005	ns
β7	Pre-vaccination CD19+ B cell count	0.4323	0.1537	0.1222 to 0.7424	2.813	0.0074	**
β8	Prevaccination $CD4^+$ T cell count	-0.7081	0.4501	-1.616 to 0.2001	1,573	0.1231	ns
β9	Prevaccination $CD8^+$ T cell count	-0.07640	0.2748	-0.6311 to 0.4783	0.2780	0.7824	ns

eTable 3. Results From Multiple Regression Analysis of Factors Affecting Anti-Spike Antibody Levels in Rituximab-Treated Patients With MS After SARS-CoV-2 Vaccination

© 2022 Tolf A et al. *JAMA Network Open*.

	0–19 B-cells/µL	20–39 B-cells/µL	>40 B-cells/µL
	n=13	n=18	n=29
**Antibody response (BAU/ml)**			
Patients with anti-spike >264 (n)	5	12	26
Percentage	38%	67%	90%
Mean anti-spike	226	564	962
SD anti-spike	252	462	709
Patients with RBD IgG >506 (n)	4	9	21
Percentage	31%	50%	72%
Mean anti-RBD	272	750	1457
SD anti-RBD	344	696	1307
**ACE2 inhibition assay (%)**			
Patients with ACE2-spike inhibition >90%			
Percentage	38%	72%	93%
Mean Inhibition ACE2-spike	72	93	96
SD Inhibition ACE2-spike	25	9	9
Patients with ACE2-RBD inhibition >90%			
Percentage	38%	67%	93%
Mean Inhibition ACE2-RBD	59	90	95
SD Inhibition ACE2-RBD	39	13	13
	**0–19 B-cells/µL**	**20–39 B-cells/µL**	**>40 B-cells/µL**
	n=10	n=17	n=25
**Patients with positive T-cell response**			
IFNg Mabtech peptide pool	10	16	22
Percentage	100%	94%	88%
IFNg in-house peptide pool	8	15	21
Percentage	80%	88%	84%

eTable 4. Humoral and Cell-Mediated Response in Different Strata of B-Cell Counts

BAU = Binding Antibody Units; IgG = immunoglobulin; anti-spike = anti-spike antibodies; anti-RBD = anti-receptor binding domain antibodies; SD = standard deviation; ACE2 = angiotensin converting enzyme 2; IFN = interferon.

© 2022 Tolf A et al. *JAMA Network Open*.
